# A Hybrid Approach for Cardiac Blood Flow Vortex Ring Identification Based on Optical Flow and Lagrangian Averaged Vorticity Deviation

**DOI:** 10.3389/fphys.2021.698405

**Published:** 2021-09-01

**Authors:** Ke Yang, Shiqian Wu, Oluwarotimi W. Samuel, Hui Zhang, Dhanjoo N. Ghista, Di Yang, Kelvin K. L. Wong

**Affiliations:** ^1^Key Laboratory of Metallurgical Equipment and Control Technology, Ministry of Education, Wuhan University of Science and Technology, Wuhan, China; ^2^Hubei Key Laboratory of Mechanical Transmission and Manufacturing Engineering, Wuhan University of Science and Technology, Wuhan, China; ^3^School of Information Science and Engineering, Wuhan University of Science and Technology, Wuhan, China; ^4^Shenzhen Institutes of Advanced Technology, Chinese Academy of Sciences, Shenzhen, China; ^5^Ultrasound Department, The Third Affiliated Hospital of Sun Yat-sen University, Guangzhou, China; ^6^University 2020 Foundation, Inc., California City, CA, United States

**Keywords:** vortex identification, Lagrangian averaged vorticity deviation, optical flow, cardiac flow analysis, vortex rings, vortex volume

## Abstract

**Objective:** The measurement of cardiac blood flow vortex characteristics can help to facilitate the analysis of blood flow dynamics that regulates heart function. However, the complexity of cardiac flow along with other physical limitations makes it difficult to adequately identify the dominant vortices in a heart chamber, which play a significant role in regulating the heart function. Although the existing vortex quantification methods can achieve this goal, there are still some shortcomings: such as low precision, and ignoring the center of the vortex without the description of vortex deformation processes. To address these problems, an optical flow Lagrangian averaged vorticity deviation (Optical flow-LAVD) method is proposed.

**Methodology:** We examined the flow within the right atrium (RA) of the participants’ hearts, by using a single set of scans pertaining to a slice at two-chamber short-axis orientation. Toward adequate extraction of the vortex ring characteristics, a novel approach driven by the Lagrangian averaged vorticity deviation (LAVD) was implemented and applied to characterize the trajectory integral associated with vorticity deviation and the spatial mean of rings, by using phase-contrast magnetic resonance imaging (PC-MRI) datasets as a case study. To interpolate the time frames between every larger discrete frame and minimize the error caused by constructing a continuous velocity field for the integral process of LAVD, we implemented the optical flow as an interpolator and introduced the backward warping as an intermediate frame synthesis basis, which is then used to generate higher quality continuous velocity fields.

**Results:** Our analytical study results showed that the proposed Optical flow-LAVD method can accurately identify vortex ring and continuous velocity fields, based on optical flow information, for yielding high reconstruction outcomes. Compared with the linear interpolation and phased-based frame interpolation methods, our proposed algorithm can generate more accurate synthesized PC-MRI.

**Conclusion:** This study has developed a novel Optical flow-LAVD model to accurately identify cardiac vortex rings, and minimize the associated errors caused by the construction of a continuous velocity field. Our paper presents a superior vortex characteristics detection method that may potentially aid the understanding of medical experts on the dynamics of blood flow within the heart.

## Introduction

The vortex formation in blood flow within the heart plays an important role in characterizing the function of the blood flow mechanism and energy transfer to the heart chamber, which are important indicators that can be used to quantify the overall heart function. The fluid flow transported by the vortex ring formation is observed to be more efficient than that transported by a steady, straight jet flow of fluid, in terms of aiding the circulation of blood to the various regions of the heart chamber (Dabiri and Gharib, 2005; Kheradvar and Gharib, 2007, 2009; Kheradvar et al., 2007; Wong et al., 2009a). As we know, the cardiac myocardium has inter-twined helical fibers. So when they contract, the heart chamber twists, and this is how blood is efficiently ejected out of the chamber. Now this heart chamber twisting causes the formation of vortex rings of high vorticity in blood flow in the chamber. In contrast, cardiomyopathic hearts have impaired contractility, and hence less twisting; hence, they have smaller vortex rings with lower vorticity (Sengupta et al., 2006). So, the formation of vortex rings is closely associated with cardiac function, and the health status of an individual. This is why, in conjunction with the structural parameters of the heart, the vortex flow analysis provides an insight into its functional analysis, and helps distinguish normal subjects from patients with heart disease (Kheradvar et al., 2019). Therefore, developing a method that can enable quantification of the vortex ring characteristics and its dynamic changes during the cardiac cycle can enable understanding of the vortex ring’s physiological functions and facilitate exploration of heart diagnostics and its pathological changes (Kräuter et al., 2020).

Interestingly, phase-contrast magnetic resonance imaging (PC-MRI) allows three-dimensional MR velocity mapping based on the intrinsic sensitivity of MRI to flow, and provides a unique tool for measuring complex blood flow patterns *in vivo* (Markl et al., 2007; Dyverfeldt et al., 2015). Earlier Wong et al. (2009a) conducted a study in which the vorticity of vortex flow was measured by using the flow field obtained from scanned PC-MRI, to characterize the location and strengths of vortices within a cardiac chamber. A major limitation of this study is that it is based on global estimation, and does not include extraction of localized information from the vortex region to elucidate the function of dominant vortexes. The identification of the dominant vortex ring is the key for the comprehensive description of the resulting swirling blood flow within a heart chamber (such as the left ventricle) resulting in blood outflow.

Since there is no uniform definition of vortex (Epps, 2017; Günther and Theisel, 2018), different vortex criteria have been used to extract vortex flow information within the heart chamber (Wong et al., 2010; Töger et al., 2012; Elbaz et al., 2014; Kräuter et al., 2020). Elbaz et al. (2014) adopted the criterion λ_2_ for left ventricular (LV) vortex detection in early and late diastolic inflow. Kräuter et al. (2020) computed the divergence-free part of the velocity vector for *Q* criterion-based identification vortex throughout the cardiac cycle. The above-mentioned methods are region-based and used to extract instantaneous vortex rings in a single frame, which hinders proper exploration of vortex formation processes. Töger et al. (2012) considered Lyapunov exponent values higher than 50% as the Lagrangian vortex ring to identify vortex boundaries. The Lagrangian vortex ring can more accurately describe the developmental process, but the method is conservative and does not include the vortex core (Kräuter et al., 2020). In Yang et al. (2021), we have employed Lagrangian averaged vorticity deviation (LAVD) to identify the cores and regions of the Lagrangian vortices and Eulerian vortices, for measuring the vortex volume and vorticity in the LV blood flow for more accurate quantification of the vortex formation. Wong et al. (2010) identified two dominant vortices of opposite rotation in the right atrium (RA), but they applied an unsupervised data clustering algorithm without considering the characteristics of blood flow. This is what has led to our preparing this paper, to improve the description of the vortex characterization in the RA by employing LAVD for more accurate identification and quantification of blood flow vortex rings.

Therefore, for accurate identification and tracking of the cardiac vortex ring characterization, this study presents a novel method (**Optical flow-LAVD**), which is precise in the extraction of Lagrangian vortex core and associated regions within the heart, based on the PC-MRI data. The proposed algorithm implements the trajectory integral of the normed deviation of vorticity from its spatial mean, for LAVD-based identification and tracking of the cardiac Lagrangian vortex rings (Haller et al., 2015). At the same time, we adopt the Horn-Schunck (Horn and Schunck, 1981) brightness constraint to synthesize the intermediate PC-MRI data for yielding high quality continuous velocity fields to reduce the error caused by the integral process of LAVD. Additionally, we have extensively validated the accuracy of the proposed Optical flow-LAVD based vortex identification method concerning synthesized PC-MRI data sequences, and we were able to characterize the region of the dominant Lagrangian vortex ring within a cardiac chamber.

## Materials and Methods

### Study Population and PC-MRI Protocol

In order to validate the performance of our proposed method of vortex ring identification and features, normal and healthy male subjects with ages around 22 years were recruited for the PC-MRI dataset collection required for the Optical flow-LAVD model’s testing. The volunteers had normal blood pressure, and no history of cardiovascular disease was observed after preliminary examination. Subsequently, we examined the flow within the RA of the participants’ hearts, by using a single set of scans pertaining to a slice at a two-chamber short-axis orientation. In this study, the RA is considered, because we can verify more accurately the effectiveness of our novel method, by referring to our previous research work on cardiac flow analysis within the atrium (Wong et al., 2009a, 2010). At the same time, our objective is to analyze the Optical flow-LAVD, and understand the developmental process of the cardiac Lagrangian vortex ring. The study was approved by the local ethical review board, and written informed consent was obtained from the subjects.

**FIGURE 1:**
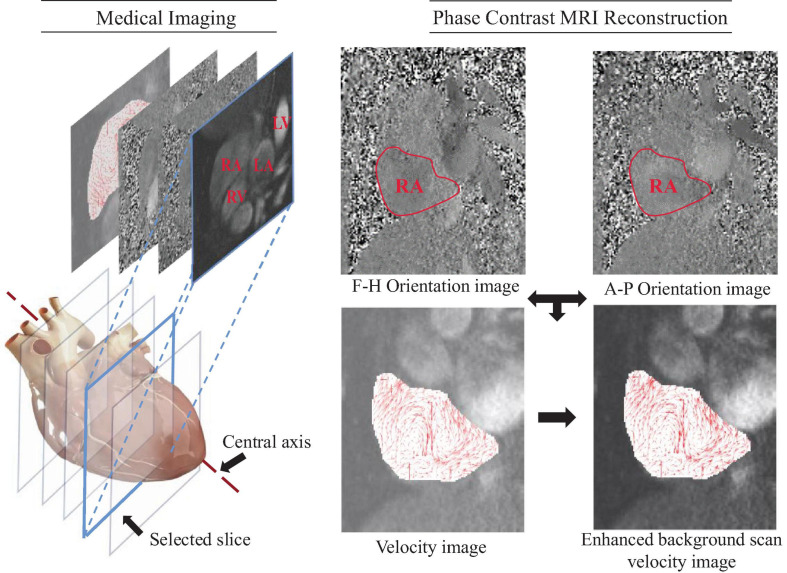
A representation of the MRI scan images through the heart, and PC-MRI based velocity images in a cardiac chamber. The PC-MRI pertains to the Foot-Head (F-H) and the Anterior-Posterior (A-P) orientations, respectively, the intensity of the whose pixels indicates the magnitude of the velocity component in the specified orientation. Combining two orthogonal velocity-encoded images can yield a velocity flow field.

The velocity-encoded magnetic resonance imaging was performed by using a Siemens Avanto, 1.5 Tesla, model-syngo MRB15 scanner with Numaris-4, Series No: 26406 software. More precisely, the encoding was set to 100 *cm*/*s* in all directions, and this configuration was applied in the case of aliasing. In addition, other parameters such as the echo time (TR) 47.1*ms*, repetition time (TE) = 1.6 *ms*, field of view (FOV) (298 = 340) *mm*^2^ at a (134 = 256) pixel matrix were configured. Further, we have employed an in-plane resolution of 1.54 *mm*/*pixel* determined by the pixel spacing, and the through-plane resolution of 6 *mm* based on the slice interval. All images were acquired with retrospective gating and 25 phases or time frames (for time frame indices from *Nt* = 1 *to* 25) for each slice, as shown in Figure 1.

### Computation of Lagrangian Average Vorticity Deviation

To identify the Lagrangian vortex ring within the right atrium (RA), the Lagrangian average vorticity deviation (LAVD) was computed in each image’s plane and consecutive time phase. Haller et al. (2015) derived the LAVD from a dynamic polar decomposition of the deformation gradient (Haller, 2016) to define vortices. The time-dependent motion trajectories of fluid particles generated by *v*(*x*, *t*) within the heart are governed by the following differential equation

(1) $$\dot{x}\left(t\right)=v\left(x,t\right),$$

defining the cardiac flow map

(2) $$\Delta {ℱ}_{t_{0}}^{t}:x_{0}\to x\left(x_{0};t\right)t\in \left[t_{o},t_{1}\right].$$

The displacement gradient of ${ℱ}_{t_{0}}^{t}$ can describe the distance of two particles from time *t*_0_ to *t*_1_, one at *x*_0_ and the other is adjacent to it at *x*_0_ + δ*x*(*t*_0_). Using mathematical representation, we have the following function

(3) $$\Delta {ℱ}_{t_{0}}^{t}\left(x_{0}\right)=\frac{dx\left(x_{0},t_{0},t\right)}{dx_{0}}t\in \left[t_{0},t_{1}\right].$$

It is to be noted that the $△{ℱ}_{t_{0}}^{t}$ tensor does not provide an objective indication of the rotational component of the deformation, because the polar rotation angle extracting from this mapping depends on the observer (Haller, 2016). To address this issue, Haller et al. (2015) used a technique based on the Dynamic polar Decomposition (DPD) to decompose ${ℱ}_{t_{0}}^{t}$ into the dynamic rotation tensor $O_{t_{0}}^{t}$ and the right dynamic stretch tensor $M_{t_{0}}^{t}$, and this is denoted as follows

(4) $$\Delta {ℱ}_{t_{0}}^{t}=O_{t_{0}}^{t}M_{t_{0}}^{t}t\in \left[t_{0},t_{1}\right].$$

Then, $O_{t_{0}}^{t}$ can be further factorized into two tensors. Specifically, we have

(5) $$\Delta {ℱ}_{t_{0}}^{t}=\Phi_{t_{0}}^{t}\Theta_{t_{0}}^{t}M_{t_{0}}^{t}t\in \left[t_{0},t_{1}\right],$$

wherein, $\Phi_{t_{0}}^{t}$ is relative rotation and $\Theta_{t_{0}}^{t}$ describes the mean rotation (Haller et al., 2015). The $\Phi_{t_{0}}^{t}$ has a dynamic consistency, which implies that the total angle swept by this tensor around its axis of rotation is dynamically consistent. Because of the feature of any physical rigid body motion, this angle will satisfy the relationship

(6) $$\psi_{t_{0}}^{t}\left(x_{0}\right)=\psi_{s}^{t}\left(x_{0}\right)+\psi_{t_{0}}^{s}\left(x_{0}\right)s,t\in \left[t_{0},t_{1}\right],$$

wherein $\psi_{t_{0}}^{t}$ is the intrinsic rotation angle. Using the result obtained in Haller (2016), it can be computed as

(7) $$\psi_{t_{0}}^{t}\left(x_{0}\right)=\frac{1}{2}\int_{t_{0}}^{t}\left|\omega \left(x\left(x_{0},s\right),s\right)-\varpi \left(s\right)\right|ds,$$

where ω(*x*(*x*_0_, *s*), *s*) is the vorticity along a material trajectory, and *ϖ* describes the spatial mean of vorticity. The LAVD is defined by an integral component of the above Eq. (7), as follows

(8) $$LAVD_{t_{0}}^{t}\left(x_{0}\right)=\int_{t_{0}}^{t}\left|\omega \left(x\left(x_{0},s\right),s\right)-\varpi \left(s\right)\right|ds.$$

Based on this Eq. (8), a rotationally coherent Lagrangian vortex is defined as a nested set of outward decreasing tubular level sets of LAVD. The LAVD is objective, and we note that this measure is relative to its neighborhood, and its value-range depends on the flow domain. In this study, the region relative to the vortex is large, and of the size of RA, based on manual segmentation. The boundary of the Lagrangian vortex is the outermost closed convex level surface of LAVD, satisfying convexity deficiency, while the vortex core is the local maxima of LAVD enclosed by the boundary (Haller et al., 2015; Yang and Melissa, 2015). Hence, we define the boundaries of Lagrangian vortex within the heart as the outermost members of closed families of LAVD level curves falling below a convexity deficiency threshold.

The LAVD also leads to a difficulty: The Lagrangian vortex is intrinsically tied to a specific finite time interval over which they exert their influence on nearby trajectories (Epps, 2017; Katsanoulis et al., 2017). Short-time variability of flow within a cardiac chamber is often seen as significant in computing the LAVD values. Therefore, we adopt the Horn-Schunck (Horn and Schunck, 1981) optical flow to synthesize the intermediate PC-MR velocity data in time and space, for producing a more accurate continuous velocity field *v*(*x*, *t*).

### Intermediate PC-MR Velocity Data Synthesis

As expressed in Eq. (8), the computation of LAVD represents an integral process, but the PC-MR velocity data are obtained within the large discrete frames and have a limited temporal resolution. So in this study, we innovatively introduce the optical flow-based intermediate PC-MR velocity data synthesis to compute LAVD.

#### Horn-Schunck Optical Flow

Recent optical flow assessment methods (Wulff et al., 2017; Xu et al., 2017) take on the variational approach introduced by Horn and Schunck (HS) (Horn and Schunck, 1981). The HS optical flow can perform on consecutive frames and predict the motion of pixels from one frame to the other, by iteratively settling an optimization issue formulated from two constraints (Afrashteh et al., 2017). The first constraint is “brightness constancy,” and it assumes that a pixel has the same brightness level in two frames after movement,

(9) I(x+u,y+v,t+dt)=I(x,y,t),

where (i) *I*(*x*, *y*, *t*) is the pixel brightness in the first frame at spatial location (*x*, *y*) and time *t*, and (ii) *I*(*x* + *u*, *y* + *v*, t + dt) is the pixel brightness in the next frame at *tdt* after (*u*, *v*) displacements from (*x*, *y*) in *x* and *y* directions, respectively. The second constraint is “spatial smoothness,” which prevents discontinuities in the flow field, as follows

(10) $${\left|\nabla u\right|}^{2}={\left(\frac{\partial u}{\partial x}\right)}^{2}+{\left(\frac{\partial u}{\partial y}\right)}^{2}and,{\left|\nabla v\right|}^{2}={\left(\frac{\partial v}{\partial x}\right)}^{2}+{\left(\frac{\partial v}{\partial y}\right)}^{2}.$$

These two constraints are combined to use the first-order form of Tikhonov’s formulation for solving the minimization problem (Horn and Schunck, 1981; Liu and Shen, 2008). Mathematically,

(11) $$\min_{u,v}\left\{\int {\left(I_{t}+I_{x}u+I_{y}v\right)}^{2}dxdy+\alpha \int \left({\left|\nabla u\right|}^{2}+{\left|\nabla v\right|}^{2}\right)dxdy\right\},$$

where (i) *I*_*t*_, *I*_*x*_, and *I_y_* are derivatives of *I*(*x*, *y*, *t*) with respect to time, spatial direction *x* and *y*, respectively, and (ii) α is the ratio of weights of the brightness constancy integral to that of spatial smoothness. This equation is solved numerically to estimate the values of *u* and *v* (Akemann et al., 2012).

#### Intermediate PC-MRI Synthesis

Given two input PC-MRI *I*_0_ and *I*_1_ and a time *t* ∈ (0, 1), our purpose is to estimate the intermediate PC-MRI $\hat{I}$ at time *T = t*. Based on HS optical flow and inspired by intermediate video frame interpolation (Meyer et al., 2015; Jiang et al., 2018), we propose fusing the warped input PC-MRI at time *T = t*. Let (*u*, *v*)_*t*→0_ and (*u*, *v*)_*t*→1_ represent the optical flow from *I_t_* to *I*_0_ and *I_t_* to *I*_1_, respectively. We can synthesize the intermediate PC-MRI $\hat{I_{t}}$ as follows

(12) $${\hat{I}}_{t}=\alpha_{0}⊙g\left(I_{0},{\left(u,v\right)}_{t\to 0}\right)\left(1-\alpha_{0}\right)⊙g\left(I_{0},{\left(u,v\right)}_{t\to 1}\right),$$

where *g*(⋅,⋅) is a backward warping function, which can be executed using bilinear interpolation and is differentiable (Zhou et al., 2016). The parameter α_0_ controls the contribution of the two input PC-MRI images, and depends on two factors: temporal consistency and occlusion reasoning (Jiang et al., 2018). The ⊙ represents element-wise multiplication, implying content perception weighting of input PC-MRI. For temporal consistency, the closer the time step *T = t* is to *T* = 0, the greater contribution of *I*_0_ makes to ${\hat{I}}_{t}$; a similar property holds for *I*_1_ (Jiang et al., 2018). On the other hand, considering the particularity of PC-MRI acquisition process, the occlusion question can be neglected. As a result, we can obtain the intermediate PC-MRI on consecutive frames based on Eq. (12) to produce a continuous velocity field for computing LAVD.

**FIGURE 2:**
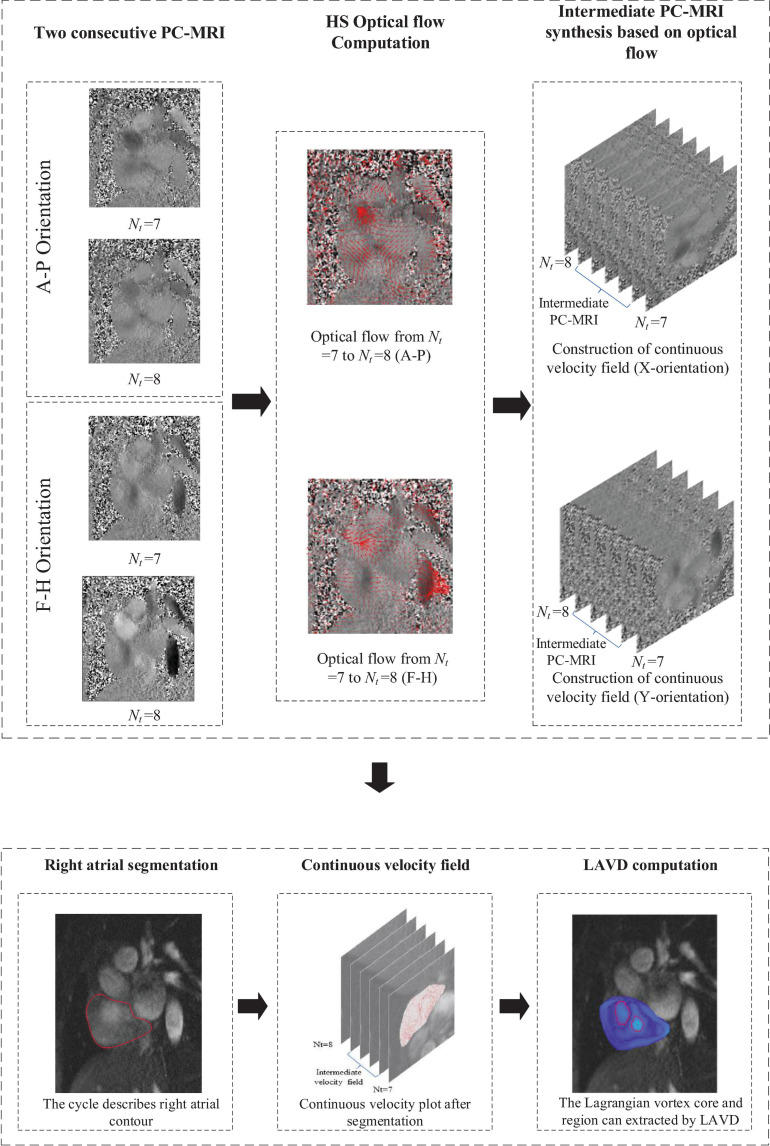
The pipeline of the proposed Optical flow-LAVD based identification of Lagrangian vortex ring.

The pipeline for Optical flow-LAVD based identification of Lagrangian vortex ring consists of four steps, and these steps are presented in Figure 2. First, the HS optical flow computation on consecutive frames is carried out. Second, based on the HS optical flow, the intermediate PC-MRI can be synthesized to produce a continuous velocity field. Third, the segmentation of the interested cardiac chamber is performed. Finally, the LAVD is computed and used to detect the Lagrangian vortex core and region within the cardiac chamber.

## Results of Analytical Studies

### Optical Flow-LAVD Based Vortex Ring Identification and Analysis

**FIGURE 3:**
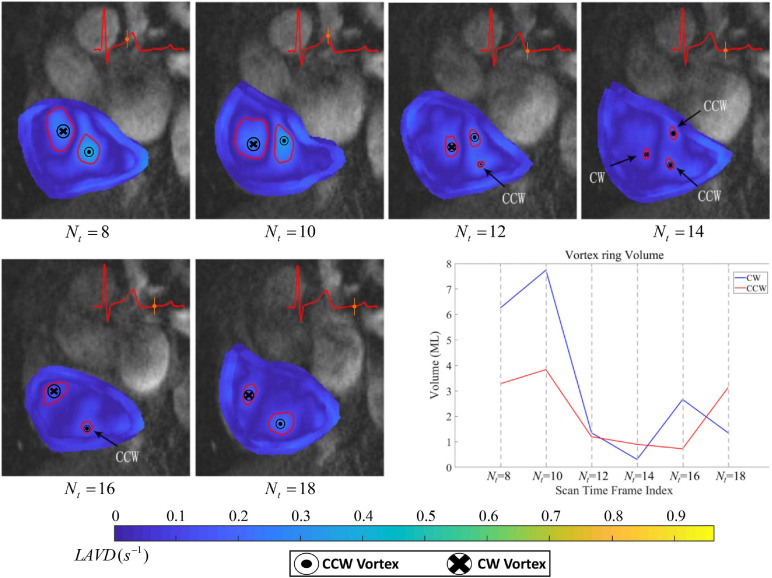
Evolution of Lagrangian vortex rings of a healthy subject. From time frame *N*_*t*_ = 8 *to* 18, the blood is swirling in both the clockwise (CW) and counter-clockwise (CCW) directions simultaneously. The LAVD contour map is produced to indicate its strength. The diagram at the lower right shows the vortex volume measured at the level of the two opposite rotation vortex rings.

The proposed algorithm successfully identifies large-scale Lagrangian vortex rings that appear in the RA and analyzes their changes and evolution during selected time frame indices from *N*_*t*_ = 8 *to* 18, As expressed in our previous research, vortex within the RA is formed and deformed during this period (Wong et al., 2009a). The variation of the Lagrangian vortex ring core and region can be visually examined by using the red circular markers as shown in Figure 3. The computation of the LAVD within the RA is based on Eq. (8), and the convexity deficiency of a closed curve in the plane is defined as the ratio of the area difference between the curve and its convex hull to the area enclosed by the curve (Haller et al., 2015). Then we can refer to our work in Yang et al. (2021) and visualized analysis, for the convexity deficiency threshold *d*_*max*_ = 1, to ensure the tubular characteristics in the heart (Yang et al., 2021). In addition, the volume of each Lagrangian vortex ring can be computed as the product of the area inside the delineation and the slice thickness, which is in line with an earlier study by Töger et al. (2012). Figure 3 illustrates the temporal evolution of vortex volume within the RA of the healthy subject.

### Horn-Schunck Optical Flow Based Intermediate PC-MRI Synthesis Analysis

#### Impact of Different Components on the Quality of PC-MRIs

For evaluation, we performed a number of ground-truth comparisons using the leave-some-out method (Meyer et al., 2015), i.e., the intermediate PC-MRIs are synthesized and compared to the original ones. We reported the Structural Similarity Index (SSIM) scores of predictions and ground-truth, as well as the interpolation errors (IE) (Meyer et al., 2015). To prevent noise and irrelevant content interference, we just computed SSIM and IE of the interested cardiac chamber.

**FIGURE 4:**
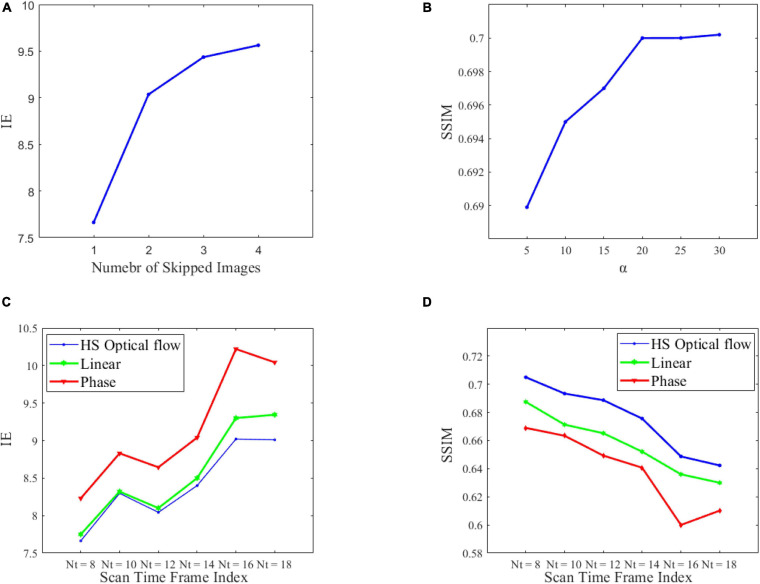
Performance of the proposed algorithm. **(A)** Quality of interpolated PC-MRI vs. the number of skipped images. **(B)** The influence of parameter α on SSIM. **(C,D)** IE and SSIM performances during the selected time frame indices for optical flow interpolation, phase-based interpolation, and linear interpolation, to deal with the construction of the intermediate PC-MRI.

We analyzed the impact of leaving out an increasing number of intermediate PC-MRI on decaying in image quality. The plot in Figure 4A visualizes the variation of IE of synthesized PC-MRI when skipping increasing frames. At the same time, we derived the SSIM and IE at different algorithm steps, as shown in Table 1. It can be seen that the optical flow computed before segmentation can obtain better intermediate images. Based on Eq. (11), we also carried out an experimental analysis of the influence of the main parameter α on the quality of reconstructed intermediate PC-MRI, as shown in Figure 4B.

**TABLE 1:** Comparison in different segmentation order.

	**IE**	**SSIM**
Before-segmentation	8.52	0.55
After-segmentation	8.29	0.67

#### Comparison With Other Interpolation Methods

We compared three different views including optical flow interpolation, phase-based interpolation, and linear interpolation to deal with the construction of the intermediate PC-MRI. The most popular methods for finding pixel correspondences across images are based on optical flow (Sun et al., 2010; Baker et al., 2011), while using image phase information directly to replace image brightness in the data term of standard optical flow formulations has been noticeable (Fleet and Jepson, 1990; Meyer et al., 2015). The phase-driven frame interpolation concept was applied to the discrete Fourier transform of an image, and it decomposes the input images into several oriented frequency bands *R*_ω,θ_ by steerable pyramid filters (Meyer et al., 2015). Then, Linear interpolation in time and space has been adopted to produce a continuous velocity field based on PC-MR velocity data (Töger et al., 2012). We have detailed the comparison of SSIM and IE during the selected time frame indices from *N*_*t*_ = 8 *to* 18, as shown in Figures 4B,C. By comparing SSIM and IE, we find that the HS optical flow method has higher prediction quality, especially in the case of existence of a large-scale vortex. Hence, combined with HS optical flow based Intermediate PC-MRI synthesis to recover a more accurate continuous velocity field, the LAVD can describe precisely the evolution of Lagrangian vortex rings within the RA, to help medical experts enhance their understanding of the physiological functions of swirling blood flow.

### Visual Analysis and the Experiment on Segmentation Dependence

**FIGURE 5:**
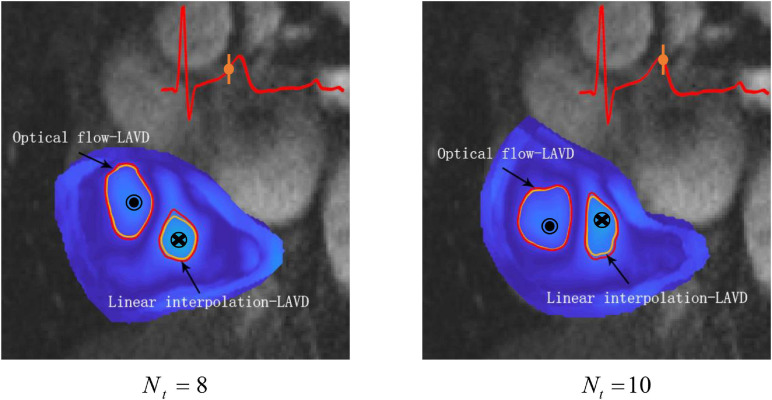
The identification results of Lagrangian vortex rings based on Optical flow-LAVD (red) and linear interpolation-LAVD (orange).

We have carried out a visualization analysis of the difference between Optical flow-LAVD and linear interpolation-LAVD, as shown in Figure 5. The identification results of vortex rings within the RA based on different methods are represented by red and orange circular markers, respectively. In order to clearly reveal the distinction of the visual results, we selected time frame indices of large-scale vortex existent from *N*_*t*_ = 8 *to* 10. It is found that the Optical flow-LAVD can more accurately describe the complexity of the Lagrangian vortex ring boundary.

In order to assess the sensitivity of Optical flow-LAVD based vortex ring extraction using different segmentation masks, we have derived the Dice similarity coefficient (DSC) as

(13) $$\frac{2TP}{TP+FP+FN},$$

where the TP, FP, TN, and FN represent the number of true positives, false positives, true negatives, and false negatives, respectively. We applied two different masks to selected time frame indices from *N*_*t*_ = 8 *to* 18 and computed the DSC of the identification results of vortex rings under manual segmentation and automatic segmentation. Table 2 provides the DSC and segmentation time to verify the robustness of the Optical flow-LAVD to different segmentation masks.

**TABLE 2:** Comparison with different segment masks.

**Method**	**DSC**	**Segmentation time (s)**
Automatic segmentation	0.89	18
Manual segmentation	1	52
		

## Discussion

We have presented a novel method for the identification of Lagrangian vortex rings core and region within the RA from PC-MRI data to allow for quantitative vortex volume. In particular, we have demonstrated (i) the variation in Lagrangian vortex rings within the RA during the diastolic phase from time frame indices *N*_*t*_ = 8 *to* 18 of one cardiac cycle with 25 phases. (ii) the characteristics of the PC-MRI by constructing accurate continuous velocity fields, based on an image-optical flow algorithm for computing LAVD.

### Lagrangian Vortex Rings Within RA Identification and Analysis

We have extracted the large-scare Lagrangian vortex rings that appear in the RA, and have analyzed their development and changes during selected time frame indices from *N*_*t*_ = 8 *to* 18, which is in accordance with the previous publications (Wong et al., 2009a,b, 2010). The development of the vortex rings core and region can be visually examined using red circular as shown in Figure 3. In addition, we superimposed the corresponding MR images onto these LAVD maps to give an indication of the location of the vortex features with respect to the chamber walls. In the beginning during time frame *N*_*t*_ = 8 *to* 10, two large-scale Lagrangian vortex rings exist in the chamber simultaneously, and we can note from Figure 3 that one counter-clockwise (CCW) Lagrangian vortex ring exists in the atrium along with a second clockwise (CW) Lagrangian vortex ring approximately to the upper-left of it. At the same time, the consequences of LAVD based identification are asymmetric, subject to the asymmetry of the heart as a whole (Kilner et al., 2000). Then later during time frame *N*_*t*_ = 12 *to* 14, two typical vortex rings are partly dissipated, and they cannot remain strictly toroid and change over time. In particular, the CCW Lagrangian vortex ring decomposes into two vortex rings of different scales, one of which moves toward the chamber wall regions. Eventually during time frame *N*_*t*_ = 16 *to* 18, we identified two dominant vortex rings of opposite rotation to grow in scale until the CCW vortex ring becomes slightly larger than the CW one.

The relative rotation region variation of two vortex rings is demonstrated by plotting the vortex volume with respect to cardiac time frames, as shown in Figure 3. Toward the end-systole from *N*_*t*_ = 8 *to* 10, the flow slows down and a corresponding dilation of the RA induces the flow in the inferior and superior *vena cava* to accelerate (Kilner et al., 2000). We note that the volume of two vortex rings of opposite rotation is increased, and the volume of the CW vortex ring and CCW vortex ring grow from 6.26 *to* 7.74 *ml* and 3.28 *to* 3.84 *ml*, respectively. With the opening of the atrioventricular valves, the blood surges into the relaxed ventricle from *N*_*t*_ = 12 *to* 14 (Kilner et al., 2000). The volume of the two vortex rings is decreased, and declines from 1.36 *to* 0.31 *ml* and 1.22 *to* 0.90 *ml* (the volume sum of two CCW vortex rings), respectively. From time frame *N*_*t*_ = 16 *to* 18, the volume of CW and CCW vortex rings change successively and range from 2.66 *to* 1.35 *ml* and 0.73 *to* 3.13 *ml*, respectively. The enlarged vortex rings can minimize stasis and thereby facilitate the flow of blood into the ventricle (Töger et al., 2012). After *N*_*t*_ = 18, we do not extract the Lagrangian vortex ring within RA, as a large amount of blood had flowed into the ventricle and there was no strict toroid flow. we set the convexity deficiency threshold to 1 in order to ensure the tubular characteristics (Haller, 2016; Yang et al., 2021). In conclusion, based on the statistics of the volume plot, we can describe the Lagrangian vortex ring development in a quantitative manner. Lagrangian vortex rings are distinctive material curves that organize the blood flow (Günther and Theisel, 2018). They present steady swirling scale flow, and manifest the persistence of Lagrangian vortex rings within the heart, to enable understanding of the role of evolution of vortices in the blood flow. We have also visualized the stretching and diffusion of vortex rings, which give deeper insight into vortex dynamics, revealing how vortex rings are weakened or reinforced by the cardiac flow field.

### Intermediate PC-MRI Synthesis Analysis

#### Impact of Different Components on the Quality of PC-MRIs

We first investigated whether leaving out an increasing number of intermediate PC-MRI decays the synthesis quality. We computed IE under four different conditions: skipping single, two, three, and four frames. Figure 4A shows the degradation in the quality of interpolated PC-MRI with increased motion between frames. In order to estimate it more reliably and accurately, we simply use two consecutive frames to construct a continuous velocity field. We have also investigated the contribution of segmentation in our method, as shown in Table 2. By applying the step of computing optical flow first and then segmentation, better interpolated PC-MRI can be acquired, which is caused by the variation of RA contours and globality of HS optical flow (Afrashteh et al., 2017). We further studied the influence of the main parameter α on the quality of interpolated PC-MRI. We can observe from Figure 4B that as the parameter α increases, the SSIM score improves. However, there is a specific threshold, such that over-sizing using larger α has little effect on reliability.

#### Comparison With Existing Interpolation Methods

We have compared the HS optical flow-based Intermediate PC-MRI synthesis method with other methods including linear interpolation (Töger et al., 2012) and phase-based interpolation (Meyer et al., 2015). They are derived from different understandings of images. The IE scores and SSIM scores on each selected time frame from three methods are shown in Figures 4C,D, and the overall evaluation is shown in Table 3. Our Optical flow-LAVD model achieves the best performance on selected time frame indices. Particularly, the HS optical flow can achieve the best IE and SSIM during the large-scale vortex existent periods from *Nt* = 8 *to* 10. In comparison, the quality of phase-based interpolated PC-MRI is slightly worse. This interpolation method captures phase information, but the irrelevant content and noise of PC-MRI have a negative effect. The variation in IE and SSIM during selected time frame indices is typically declining. This is due to larger pixel displacement and weakly turbulent flow pattern during the end of RA diastole period (Kheradvar et al., 2019).

**TABLE 3:** Comparisons with different interpolation methods.

	**IE**	**SSIM**
HS optical flow interpolation	**8.29**	**0.67**
Linear interpolation	8.56	0.65
Phased-based interpolation	9.17	0.62

*Bold values highlight the highest scores.*

### Visual Analysis and Segmentation Dependence

Between the Optical flow-LAVD and linear interpolation-LAVD, visual analysis finds the differences in their Lagrangian vortex ring identification results. During the large-scale vortex existence period, the Optical flow-LAVD detected vortex ring region is wide and its boundary fits a complex ellipse, especially in the case of CW vortex rings shown in Figure 5. It can be found that the Optical flow-LAVD can more realistically describe the Lagrangian vortex rings formation. Furthermore, the computation of LAVD is an integral process. Two different vortex ring identification results boil down to the accumulation of numerical integration errors, which also indicates the importance of the high-performance interpolation method.

Regarding the vortex ring identification results under the manual segmentation as the reference condition, we compute the overlap of vortex regions under automatic segmentation and compare the segmentation time. The automatic segmentation masks are seen to yield poor agreement for Lagrangian vortex ring extraction, as shown in Table 2, which demonstrates the sensitivity of Optical flow-LAVD when using different segmentation approaches. While the LAVD is objective, we note that this measure is relative to its neighborhood, and its value-range depends on the neighborhood size (Günther and Theisel, 2018). Thus, the precision of automatic segmentation to achieve our algorithm speed-up is needed.

In summary, the HS optical flow-based interpolation method achieves the best results over the PC-MRI sequence, in generating a more accurate continuous velocity field. This is remarkable and interesting, Considering the characteristics of the PC-MRI, we combine the image-interpolation algorithm to compute the Lagrangian features of cardiovascular blood flow.

### Limitations

There are several works that need to be improved in the proposed purposed method. Firstly, no reference standard exists for Lagrangian vortex rings within the cardiac chambers. However, the numerical simulation model we used is in excellent agreement with the model of Haller et al. (2015). There are no comparison experiments between healthy controls and patients. Nevertheless, the focus of this study is to provide a proof of concept of Optical flow-LAVD based vortex ring identification and, as discussed previously, combining optical flow can improve the accuracy of describing vortex evolution. In our following investigations, we will consider obtaining the synthetic data based on fluid dynamics simulations (Rispoli et al., 2015; Rajat et al., 2016)and diagnosis of additional pathological patients, in order to further validate the performance of our method, based on Optical Flow-LAVD. Vortices within the ventricular chambers reflect the pathophysiological link between the diastolic filling and systolic ejection, and present a better reproducibility because of their higher intensity field (Hong et al., 2013; Kheradvar et al., 2019). For that purpose, identification of vortices within the ventricular chambers by Optical flow-LAVD is to be the focus of our future work. Secondly, the blood flow in the heart is accompanied by the formation of vortex rings which dynamically occupy the 3D space of the heart chamber (Kräuter et al., 2020). The 3D vortex ring extraction is significant for a more accurate description of vortex formation (Wong et al., 2009a). In fact, the computation of LAVD to identify the 3D vortex ring in the heart has not been investigated, and remains a topic for our future research. Thirdly, we have strictly set the convexity deficiency threshold to ensure the tubular characteristics, leading to ignoring some vortices with a relative ring shape (Yang et al., 2021). So, future work is required to establish an adaptive method instead of setting threshold artificially. We also recognize that LAVD is relative to its neighborhood, and its value-range depends on the neighborhood size (Günther and Theisel, 2018). Thus, a high demand is placed on the method of high-precision segmentation of the heart chamber. Finally, we have used the HS optical flow-based interpolation method to achieve the best results over the PC-MRI sequence. However, the IE scores and SSIM scores on each selected time frame highlight the insufficiency of the interpolation model’s capacity to deal with the challenging motion regions of the heart; the temporal sampling in medical image sequences are lower than that of natural scene videos (Guo et al., 2020; Samuel et al., 2020), and the computation of optical flow is expensive (Meyer et al., 2015; Samuel et al., 2017). Improving the optical flow-based interpolation performance, and decreasing the computation time are also key factors for our future research.

## Conclusion

In this study, we have proposed a novel cardiac Lagrangian vortex rings identification method, based on a combination of LAVD and optical flow. We first estimate the optical flow between two consecutive PC-MRI, and the intermediate optical flow fields can be approximated by warping the procedure for constructing continuous velocity fields. We then compute LAVD by using the synthesized velocity field to accurately extract the Lagrangian vortex core and region within RA. The temporal evolution of Lagrangian vortex rings within RA is described for the first time; it is found that the optical flow-based interpolation method achieves the best results over the PC-MRI sequence. Our method provides a solution for the cardiac vortex ring formation analysis, and improves understanding of blood flow dynamics within the heart.

## Data Availability Statement

The raw data supporting the conclusions of this article will be made available by the authors, without undue reservation.

## Ethics Statement

The studies involving human participants were reviewed and approved by the Royal Adelaide Hospital Committee and the Institutional Review Board. The patients/participants provided their written informed consent to participate in this study.

## Author Contributions

KY, SW, OS, HZ, DG, DY, and KW processed the data for analysis and performed the statistical analysis. All authors contributed to the study design, data interpretation, and writing of the report.

## Conflict of Interest

DG was employed by company University 2020 Foundation, Inc. The remaining authors declare that the research was conducted in the absence of any commercial or financial relationships that could be construed as a potential conflict of interest.

## Publisher’s Note

All claims expressed in this article are solely those of the authors and do not necessarily represent those of their affiliated organizations, or those of the publisher, the editors and the reviewers. Any product that may be evaluated in this article, or claim that may be made by its manufacturer, is not guaranteed or endorsed by the publisher.

## References

[B1] Afrashteh N. Inayat S. Mohsenvand M. Mohajerani M. H. (2017). Optical-flow analysis toolbox for characterization of spatiotemporal dynamics in mesoscale optical imaging of brain activity. *Neuroimage* 153 58–74. 10.1016/j.neuroimage.2017.03.034 28351691

[B2] Akemann W. Mutoh H. Perron A. Park Y. K. Iwamoto Y. Knöpfel T. (2012). Imaging neural circuit dynamics with a voltage-sensitive fluorescent protein. *J. Neurophysiol.* 108 2323–2337. 10.1152/jn.00452.2012 22815406

[B3] Baker S. Scharstein D. Lewis J. P. Roth S. Black M. J. Szeliski R. (2011). A database and evaluation methodology for optical flow. *Int. J. Comput. Vis.* 92 1–31. 10.1007/s11263-010-0390-2

[B4] Dabiri J. O. Gharib M. (2005). The role of optimal vortex formation in biological fluid transport. *Proc. Biol. Sci.* 272 1557–1560. 10.1098/rspb.2005.3109 16048770 PMC1559837

[B5] Dyverfeldt P. Bissell M. Barker A. J. Bolger A. F. Carlhäll C. J. Ebbers T. (2015). 4D flow cardiovascular magnetic resonance consensus statement. *Cardiovasc. Magn. Reson.* 17:72. 10.1186/s12968-015-0174-5 PMC4530492 26257141

[B6] Elbaz M. S. M. Calkoen E. E. Westenberg J. J. M. Lelieveldt B. P. F. Wroest A. A. Geest R. (2014). Vortex flow during early and late left ventricular filling in normal subjects: quantitative characterization using retrospectively-gated 4D flow cardiovascular magnetic resonance and three-dimensional vortex core analysis. *J. Cardiovasc. Magn. Reson.* 16:78. 25270083 10.1186/s12968-014-0078-9 PMC4177574

[B7] Epps B. (2017). “Review of vortex identification methods,” in *Proceedings of the 55th AIAA Aerospace Sciences Meeting*, (Grapevine, TX), 989–1001.

[B8] Fleet D. J. Jepson A. D. (1990). Computation of component image velocity from local phase information. *Int. J. Comput. Vis.* 5 77–104. 10.1007/bf00056772

[B9] Günther T. Theisel H. (2018). The state of the art in vortex extraction. *Comput. Graph. Forum* 37 149–173. 10.1111/cgf.13319

[B10] Guo Y. Y. Lie B. Ahn E. Feng D. Wang Q. Kim J. (2020). “A spatiotemporal volumetric interpolation network for 4D dynamic medical image,” in *Proceedings of the 2020 IEEE/CVF Conference on Computer Vision and Pattern Recognition*, (Seattle, WA), 4725–4734.

[B11] Haller G. (2016). Dynamic rotation and stretch tensors from a dynamic polar decomposition. *J. Mech. Phys. Solids* 86 70–93. 10.1016/j.jmps.2015.10.002

[B12] Haller G. Hadjighasem A. Farazmand M. Huhn F. (2015). Defining coherent vortices objectively from the vorticity. *J. Fluid Mech.* 795 136–173. 10.1017/jfm.2016.151

[B13] Hong G. R. Kim M. Pedrizzetti G. Vannan M. A. (2013). Current clinical application of intracardiac flow analysis using echocardiography. *J. Cardiovasc. Ultrasound* 21 155–162. 10.4250/jcu.2013.21.4.155 24459561 PMC3894365

[B14] Horn B. K. Schunck B. G. (1981). Determining optical flow. *Artif. Intell.* 17 185–204.

[B15] Jiang H. Z. Sun D. Q. Jampani V. Yang M. H. Miller E. L. Kautz J. (2018). “Super SloMo: high quality estimation of multiple intermediate frames for video interpolation,” in *Proceedings of the 2018 IEEE/CVF Conference on Computer Vision and Pattern Recognition* (Salt Lake City, Hawaii: Hawaii), 9000–9008.

[B16] Katsanoulis S. Farazmand M. Serra M. Haller G. (2017). Vortex boundaries as barriers to diffusive vorticity transport in two-dimensional flows. *Phys. Rev. Fluids* 5:24701.

[B17] Kheradvar A. Gharib M. (2007). Influence of ventricular pressure drop on mitral annulus dynamics through the process of vortex ring formation. *Ann. Biomed. Eng.* 35 2050–2064. 10.1007/s10439-007-9382-y 17899379

[B18] Kheradvar A. Gharib M. (2009). On mitral valve dynamics and its connection to early diastolic flow. *Ann. Biomed. Eng.* 37 1–13. 10.1007/s10439-008-9588-7 18982451

[B19] Kheradvar A. Milano M. Gharib M. (2007). Correlation between vortex ring formation and mitral annulus dynamics during ventricular rapid filling. *ASAIO J.* 53 8–16. 10.1097/01.mat.0000249870.44625.22 17237643

[B20] Kheradvar A. Rickers C. Morisawa D. Kim M. Hong G. R. Pedrizzetti G. (2019). Diagnostic and prognostic significance of cardiovascular vortex formation. *J. Cardiol.* 74 403–411. 10.1016/j.jjcc.2019.05.005 31255458

[B21] Kilner P. J. Yang G. Z. Wilkes A. J. Mohiaddin R. H. Firmin D. N. Yacoub M. H. (2000). Asymmetric redirection of flow through the heart. *Nature* 404 759–761. 10.1038/35008075 10783888

[B22] Kräuter C. Reiter U. Reiter C. Nizhnikava V. Masana M. Schmidt A. (2020). Automated mitral valve vortex ring extraction from 4D-flow MRI. *Magn. Reson. Med.* 84 3396–3408. 10.1002/mrm.28361 32557819 PMC7540523

[B23] Liu T. Shen L. (2008). Fluid flow and optical flow. *J. Fluid Mech.* 614 253–291. 10.1017/s0022112008003273

[B24] Markl M. Harloff A. Bley T. A. Zaitsev M. Jung B. Weigang E. (2007). Time-resolved 3D MR velocity mapping at 3T: improved navigator-gated assessment of vascular anatomy and blood flow. *Magn. Reson. Imaging* 25 824–831. 10.1002/jmri.20871 17345635

[B25] Meyer S. Wang O. Zimmer H. Grosse M. Hornung A. S. (2015). “Phase-based frame interpolation for video,” in *Proceedings of the 2015 IEEE Conference on Computer Vision and Pattern Recognition*, (Boston, MA), 1410–1418.

[B26] Rajat M. Seo J. H. Vedula V. Choi Y. J. Liu H. Huang H. H. (2016). Computational modeling of cardiac hemodynamics: current status and future outlook. *J. Comput. Phys.* 305 1065–1082.

[B27] Rispoli V. C. Nielsen J. F. Nayak K. S. Carvalho J. L. A. (2015). Computational fluid dynamics simulations of blood flow regularized by 3D phase contrast MRI. *BioMed. Eng. OnLine* 14:110. 26611470 10.1186/s12938-015-0104-7 PMC4661988

[B28] Samuel O. W. Asogbon G. M. Sangaiah A. K. Fang P. Li G. (2017). An integrated decision support system based on ANN and Fuzzy_AHP for heart failure risk prediction. *Expert Syst. Appl.* 68 163–172. 10.1016/j.eswa.2016.10.020

[B29] Samuel O. W. Yang B. Geng Y. Asogbon M. G. Pirbhulal S. Mzurikwao D. (2020). A new technique for the prediction of heart failure risk driven by hierarchical neighborhood component-based learning and adaptive multi-layer networks. *Future Gener. Comput. Syst.* 110 781–794. 10.1016/j.future.2019.10.034

[B30] Sengupta P. P. Korinek J. Jahangir A. (2006). Left ventricular structure and function: basic science for cardiac imaging. *J. Am. Coll. Cardiol.* 48 1988–2001. 17112989 10.1016/j.jacc.2006.08.030

[B31] Sun D. Roth S. Black M. J. (2010). “Secrets of optical flow estimation and their principles,” in *Proceedings of the 2010 IEEE Computer Society Conference on Computer Vision and Pattern Recognition*, (San Francisco, CA), 2432–2439.

[B32] Töger J. Kanski M. Carlsson M. Kovacs S. J. Söderlind G. Arheden H. (2012). Vortex ring formation in the left ventricle of the heart: analysis by 4d flow mri and lagrangian coherent structures. *Ann. Biomed. Eng.* 40 2652–2662. 10.1007/s10439-012-0615-3 22805980

[B33] Wong K. K. L. Kelso R. M. Worthley S. G. Sanders P. Mazumdar J. Abbott D. (2009a). Cardiac flow analysis applied to phase contrast magnetic resonance imaging of the heart. *Ann. Biomed. Eng.* 37 1495–1515. 10.1007/s10439-009-9709-y 19466548

[B34] Wong K. K. L. Kelso R. M. Worthley S. G. Sanders P. Mazumdar J. Abbott D. (2009b). Theory and validation of magnetic resonance fluid motion estimation using intensity flow data. *PLoS One* 4:e4747. 10.1371/journal.pone.0004747 19270756 PMC2651647

[B35] Wong K. K. L. Tu J. Kelso R. M. Worthley S. G. Sanders P. Mazumdar J. (2010). Cardiac flow component analysis. *Med. Eng. Phys.* 32 174–188. 10.1016/j.medengphy.2009.11.007 20022796

[B36] Wulff J. Sevilla-Lara L. Black M. J. (2017). “Optical flow in mostly rigid scenes,” in *Proceedings of the 2017 IEEE Conference on Computer Vision and Pattern Recognition* (Salt Lake City, Hawaii: Hawaii), 6911–6920.

[B37] Xu J. Ranftl R. Koltun V. (2017). “Accurate optical flow via direct cost volume processing,” in *Proceedings of the 2017 IEEE Conference on Computer Vision and Pattern Recognition* (Salt Lake City, Hawaii: Hawaii), 5807–5815.

[B38] Yang K. Wu S. Zhang H. Ghista D. N. Samuel O. W. Wong K. K. L. (2021). Lagrangian-averaged vorticity deviation of spiraling blood flow in the heart during isovolumic contraction and ejection phases. *Med. Biol. Eng. Comput.* 59 1417–1430. 10.1007/s11517-021-02366-2 34115272

[B39] Yang Z. H. Melissa A. G. (2015). “Comparing leading and trailing edge vortex circulation history with vortex identification and tracking methods,” in *Proceedings of the 54th AIAA Aerospace Sciences Meeting*, (San Diego, CA), 2082–2092.

[B40] Zhou T. Tulsiani S. Sun W. Malik J. Efros A. A. (2016). “View synthesis by appearance flow,” in *Computer Vision – European Conference on Computer Vision 2016. Lecture Notes in Computer Science*, Vol. 9908 eds Leibe B. Matas J. Sebe N. Welling M. (Cham: Springer), 286–301. 10.1007/978-3-319-46493-0_18

